# Dental Education in Germany

**Published:** 1888-02

**Authors:** W. D. Miller

**Affiliations:** Berlin


### DENTAL EDUCATION IN GERMANY.

BY W. D. MILLER, BERLIN.

CONCLUDED FROM PAGE 11.

Many German Dentists have, in the past, gone to America to pursue their professional studies and to obtain their doctor title. It would be very unjust to suppose all such to be quacks, for many of them are men of good standing, with large practices, and this they claim is one reason why the American degree is so cordially detested by the Zahnärzte.

The centre of dental education in Germany lies at present in Berlin, and the progress of the Dental Institute, which was established as a department of the Royal University in 1884, has been watched with great interest. At first the institute was looked upon as an experiment; now it can be recognized as wonderful success, and the impulse it has given to the study of dentistry in Germany is no less wonderful. Previous to the establishment of the Dental Institute in Berlin, the number of students of dentistry at the University averaged about thirty-five; some years it was as low as twenty. I am also informed that the courses and lectures conducted by Prof. Albrecht, who, for many years had charge of the dental polyclinic, were attended by very few students, and sometimes only half a dozen hearers would be present, very seldom but two or three. The professor would then sit down by them on the bench and give his lecture in the form of a quiet little chat. The accommodations in the clinic itself, were almost *nil*. The number of students matriculated in 1883 was forty; in 1884 it immediately rose to sixty, and last semester it had risen to 147 ; this semester the number has not yet been made known. I estimate it at about 165. The number of students who took the operating course, alone, the first year in the dental institute, was fifteen. This number has gradually increased, till in the present semester it has reached fifty-eight, which is the highest possible number that we can accommodate. Many more applied, but we were obliged to refuse the applications, simply for want of room. The increase in the other departments has been equally great. The work done at the dental institute is divided into three departments :

(1). Extractions and minor surgical operations, in charge of Prof. Dr. Busch.

(2). Conservative treatment of the teeth, in charge of Profs. Drs. Miller and Poetsch.

(3). Mechanical dentistry, in charge of Prof. Saer.

The conditions for admission to the study of dentistry are those stated above, viz : Reife für Prima in a German gymnasium, or first-class Real Schule. With this certificate students are matriculated for four semesters in the philosophical faculty of the University. Those who desire to study more than four semesters must, before the end of this time, apply to the Curatorium of the University, and receive the privilege of studying two semesters longer.

The charges for matriculation are very small—about five dollars—but each course must be paid for separately. The course in extraction costs about twelve dollars, that in operative dentistry the same, while mechanicl dentistry costs thirty dollars. A course of lectures, three per week, without demonstrations, cost five dollars.

The clinic conducted by Prof. Busch is, nominally, daily, from 11 to 1 o'clock, but it is very seldom that the material is finished at 1 o'clock. The work done is principally extraction, as Prof. Busch does not encourage the students in the veiw that the dentist should also be capable of performing resections of the jaw, removing cancer and operations of that nature. In this he is undoubtedly right. These operations belong in the domain of surgery, and no person who has not had a thorough surgical education has any business to trifle with them. Consequently, besides extractions, the operations performed are chiefly incisions of abscesses, treatment of diseases of the antrum, of cysts, and minor operations of that kind. Prof. Busch is himself present the whole time, and personally superintends each operation.

The clinic for extraction presents a marked contrast to that in most colleges in America. It is sought daily by about forty to fifty patients from the lowest classes, the number of teeth extracted daily averaging about seventy-five. Three or four narcoses are made daily, almost exclusively with nitrous oxide. Some ten or twelve extractions were made under cocaine-anæsthesia. In every case, however, the results were unsatisfactory, so that this anæsthetic has been abandoned. The operating rooms are open daily from one to six in summer, and from twelve till dark in winter. The rooms accommodate, with difficulty, twenty-eight chairs, and each chair is occupied by two students, who can arrange between themselves as to the hours in which each is to operate. There is usually an abundance of material, so that on some days all of the twenty-eight chairs are occupied, and the rooms present an aspect of greatest activity. I myself am always present on Monday, Tuesday and Wednesday, from two to four, and Prof. Paetsch at the same hours on Thursday, Friday and Saturday. One assistant and one demonstrator are daily present. Both of the professors, as well as the assistant and demonstrator, do a great deal of work in the clinic, so that each student has repeated opportunities of seeing various operations performed, as well as receiving directions during the performance of the operations himself. We endeavor to give as thorough instruction as is possible in the time allotted, in filling with non-cohesive as well as with cohesive gold (pellets, cylinders and foil), with tin and gold, and with the various plastics; much attention is also given to the conservative treatment of the pulp, and to the antiseptic treatment of root canals. I think I may say, with all impartiality and proper respect for the institutions of my fatherland, that the instruction here received by the students quite as well qualifies them *for the practice of dentistry in Germany* as that which they would receive in an equal length of time in any dental institution of America.

In educating a man for any occupation some regard must be had to the circumstances under which he is to carry out the same. A young man brought up in the belief that everything must be done with gold, and that, too, with "hard gold," and that in every case the tooth must be restored to its original shape, and that the gold must have exactly the same density throughout, and a surface as bright as a mirror, *might* make a living in New York, or some of the larger American cities, but if he were put into a German city of 10,000 to 40,000 inhabitants, I doubt very much if he would be able to collect a practice. The great majority of the students from the dental institute set up in practice in cities where it is absolutely necessary for them to make many operations in plastic materials, and where, if they do gold work, they must do it at inferior prices, and where they will be obliged to sacrifice to the forceps many teeth which could be saved. The mass of the German people must first be taught the value of good conservative dentistry, before they will put any confidence in it, and before they will be willing to pay for it, and this teaching is not a matter of a few days, but of many years, and they must learn by *experience* that fillings can be made which will not fall out the next day, and that it is not necessary to extract a tooth as soon as it becomes carious.

The extension of the freedom to practice dentistry to all, regardless of qualification, and the too little attention paid to the practical side of the education of the Zahnärzte, has been a great hinderance to the growth of conservative dentistry in Germany. The many dabblers in dentistry, Zahnartists, Zahnkünstler, Tooth-healers, etc., all over Germany, as well as many of the Zahnärzte, who, as far as their dental education is concerned are little more than Zahntechniker, have for many years been inculcating by their practice a lesson which can be counteracted only by years of successfull practice in the conservation of the teeth. While we, therefore, endeavor to prepare our students for conducting a first-class practice, they must also be able to conduct second-class practices; *i. e.*, practices among the less wealthy classes of people, and among people who have for centuries been brought up in the belief that there is little to be expected of conservative treatment, and who do not readily "catch on to new notions." We are, therefore, obliged to give our students a broader education than would be necessary for many a practice in America, and our system of teaching, instead of producing the one-sided results which Dr. Abbott thought he might attribute to us, produces beyond doubt a more many-sided preparation than is obtained in the majority of American schools.

Every student learns to extract under the eyes and hands of the instructor. He extracts during his study many teeth himself, and sees thousands extracted. He witnesses hundreds of narcoses with $N\ O_2$ (some also with other anæsthetics), *must himself be perfectly competent to conduct the narcosis,* has the opportunity of seeing perhaps all the diseases which the human mouth is heir to, and learns to diagnose them and operate upon such as it is desirable for the dentist to operate upon. He learns to use all the filling materials that have been found of value in dentistry; the process is demonstrated to each student separately; he must also be equally well prepared to diagnose the most important diseases of the dental pulp, and treat them accordingly; he must thoroughly understand the principles and practice of the antiseptic treatment of pulpless teeth, etc., etc. In mechanical dentistry a proportionate amount of time is spent. The student is not allowed to take a patient, pick out the places where he can insert showy gold fillings and dismiss the patient with the others untouched. He must *put the mouth in order,* and a control is kept of all the work done. The difficulty of our work is increased by the fact that we must begin with the A B C. The majority of students entering an American dental college are already able to insert a fair gold filling; here it is the exception that the beginner knows anything at all about the manipulation of gold or the preparation of cavities.

We have no clinics in the American sense of the word, and this for various reasons. (1) At the time of the founding of the institute one could have counted its friends among the Zahänrzte upon the fingers of one hand, and probably still have some fingers over. It would have been somewhat difficult to secure good operators for clinics, if it had been deemed desirable. (2) Our students are obliged to do a great deal of work in the four semesters, and the number of chairs being limited we would not feel justified in closing the rooms for the entire day, and thus depriving all the twenty-eight students of the much-needed exercise at the chair. All the more, as I have serious doubts as to the value of clinics for students. It is, or should be, the aim of a course of operative dentistry to instruct the student in the general or fundamental principles pertaining to the practice of dentistry. He should learn how to form the cavities in such a manner as to give the best form for retaining its filling without weakening the tooth or sacrificing an unnecessary amount of substance. He should thoroughly comprehend the properties and manipulation of cohesive as well as of non-cohesive gold, and of plastic materials, and be able to determine, according to the conditions, which material is best adapted for individual cases. He should be familiar with the predisposing as well as the direct causes of decay of the teeth, and those conditions in the human mouth which favor the appearance of decay, so as to be able, as far as possible, to obliterate them by his operation on the teeth. He should exercise himself in acquiring a delicate touch and a firm steady hand, which is half the battle in the treatment of sensitive teeth, exposed pulps and root canals. He should understand thoroughly the character of the material he is operating upon, and the objects which it is desired to secure by the operation, etc., etc. In other words, he must be taught the fundamental principles which should be observed in the treatment of every tooth, no matter what material or which method is employed. These things he will learn better by working two or three hours himself, under the eyes of the teacher, than by spending an equal time in watching some one else condensing a certain amount of gold, often in a previously prepared cavity, the operator, perhaps, being too much engrossed with his operation to be able to give the necessary explanations which should accompany every demonstration.

I doubt, on the whole, from what I have seen of these clinics, whether the students are much benefited by them, and when they are not compelled to attend but a small per cent. put in their appearance, even at the beginning of the operation, and before it is over, as a rule, only half a dozen, sometimes only two or three, will be left. For practitioners, as well as for students who do not daily receive *practical* instruction in filling from the teacher, I hold clinics to be of considerable value, but our experience thus far has been such that we do not feel inclined to change our present system, or to introduce the clinic system in vogue in most of the American dental colleges. Nevertheless, if any one has a new method, new instrument, or a new operation which he wishes to demonstrate, he is allowed to do so. He is given a chair and a person to work upon. Those students who are free and wish to can look on, but no one is compelled to stop work, put up his instruments and lose a day's work on that account.

Examinations do not take place here at the close of each winter semester, as in America, but at any time between November 1st and about August 15th, when three or four students, who have studied the required four semesters and have the preliminary qualification (Reife für Prima), present a formal request for admission. The examinations are divided into four stations or stages. In the first, the candidate is confronted with a patient from the polyclinic, and is required to examine the mouth and teeth and to diagnose the cause of any trouble of which the patient may complain; also to describe the character of and give treatment for any diseased condition of the teeth or associate parts which may be present. He is examined orally for thirty minutes by two members of the examination committee, and then commits the whole matter to writing.

In the second station, the examination is written only. Here the candidate draws ten questions from a box containing 150. The questions are usually very comprehensive, so that ten, twelve, or even fourteen hours are consumed in writing the answers to them, the examination beginning at 9 A. M., and lasting sometimes till 11 P. M.

The questions are on Toxicology, Materia Medica, Anatomy (general and special), Physiology, Pathology, Therapeutics. The following are some of the questions:

(1.) Where do fractures occur in the upper jaw? what are the symptoms? how are fractures occasioned? how are they treated?

(2.) What irregularities are observed of individual teeth? what are the causes? what are the disturbances and diseases produced by them? how are they prevented and removed?

(3.) Nervous Trigeminus; trace the course of all its branches.

(4.) Describe the structure of the mucous membrane of the mouth in general, and of the gums in particular.

(5.) Mercury—its chemical and pharmaceutical preparations.

Ten questions of this character are not quickly disposed of. The list of questions is very old, and the candidates know pretty well what they are. This matters little, however, since if they thoroughly work up 150 questions of this kind, they deserve to pass. Nevertheless, we hope to be able to prepare a new list before long. In the third station, the candidates are examined in the conservative treatment of the teeth (Miller), extracting (Busch), mechanical dentistry (Baume).

As for the examination in the conservative treatment of the teeth, its severity depends altogether upon my knowledge of the candidate.

If I have convinced myself, during the months that he has worked under my charge, that he is a capable operator, it is made very easy; if I have any doubt about his efficiency, it will naturally be made more difficult. He is obliged to make one or more operations of any nature that I may see fit to require, directly under my eyes. It may be a filling or a pulp treatment, or treatment of root-canal, abscessed root, etc., etc.

He must also perform a number of difficult extractions under Prof. Busch. Thirdly, he makes a set of teeth on rubber, a gold clasp and a pivot tooth in the laboratory of and under the eye of Dr. Baume; the last two are made on the model, not in the mouth. In the fourth station (Schluss, conclusion), he is examined orally by three examiners (one-quarter hour each) on anatomy in particular, and on any subject in practical or scientific dentistry. The marks given by the individual examiners are: (1) Very good; (2) Good; (3) Sufficient; (4) Insufficient; (5) Bad.

The mark 4 (of course also five) obtained from any one examiner in any station throws the candidate out. He can, however, try again after a period of six to twelve weeks. The shortest time in which all the stations can be completed is about two weeks.

It will be seen from the above that the examination is rather a complicated affair, and by no means very easy. During the last winter, out of the twenty-nine candidates eight were rejected in one or more of the stations. If some of the dental colleges of America would approach these figures, the effect would be very salutary to the standing of the profession.

The requirements are being gradually raised in Berlin, so that many of our less energetic students make their studies in Berlin, and then go to some other university to pass the examinations. The examination is not a university or institute examination, but a State examination, and is conducted by a board of examiners appointed each year by the State, who do not necessarily have any connection with the University.

The present examining board consists of Privy Councillor Prof. Dr. Waldeyer, (Anatomy); Prof. Dr. Busch, (Surgery); Prof. Dr. Miller, (Operative Dentistry); Prof. Dr. Baume, (Mechanical Dentistry).

The examiners do not, however, confine themselves strictly to the branches placed opposite their names. The candidate who has successfully passed through all the stations receives a diploma, conferring upon him the title "Approbirter Zahnarzt," or "Praktischer Zahnarzt" (approved tooth-physician or practicing tooth-physician). It is at present only a title, and confers upon its possessor no particular immunity except a better standing in society, the right to prescribe medicines, administer anæsthetics, etc.

Regarding education in general, and dental education in particular, Dr. Abbott made some statements at the last meeting of the American Dental Association (see INDEPENDENT PRACTITIONER, September, p. 482), which ought not to pass unnoticed. I will, however, only say that the statements referred to are entirely wrong, and that any one who wishes to inform himself on matters of education in Germany would do well to look to some other source than the one referred to. It is not possible to establish a dental school in a place where practical dentistry is not far advanced, and bring it to perfection in three years. There is much room for improvement; nevertheless, every one who has visited our school has been astonished at what we have already accomplished. Dr. Harlan, who visited the Institute a year and a half ago, says that the operations were "quite equal to the average in our country," *i. e.*, in America, which, considering the difficulties with which we have had to contend is saying a great deal. Our students do not perform as extensive operations as are often indulged in by the students in American colleges, but in the way of care, exactness and thoroughness in preparing the cavity and inserting the material, we have had students whose work, I think, very nearly, if I may not say quite, equaled anything that I have seen done by the students in America. These results, I am inclined to think, are largely to be attributed to the methods of teaching here in vogue, and to the individual efforts of the teachers in this department.

As to whether it would be expedient to increase the requirements for admission, as has been talked of, or to demand for admission to American colleges a general education equivalent to that now required here, is a question on which I may offer my views at another time.

Also the recent dental legislation in Germany, the warfare upon the Dr. title (D. D. S.), and the standing of various American dental colleges in Germany may receive attention later.

